# Model storage, exchange and integration

**DOI:** 10.1186/1471-2202-7-S1-S11

**Published:** 2006-10-30

**Authors:** Nicolas Le Novère

**Affiliations:** 1 EMBL-EBI, Wellcome-Trust Genome Campus, Hinxton, UK

## Abstract

The field of Computational Systems Neurobiology is maturing quickly. If one wants it to fulfil its central role in the new Integrative Neurobiology, the reuse of quantitative models needs to be facilitated. The community has to develop standards and guidelines in order to maximise the diffusion of its scientific production, but also to render it more trustworthy. In the recent years, various projects tackled the problems of the syntax and semantics of quantitative models. More recently the international initiative BioModels.net launched three projects: (1) MIRIAM is a standard to curate and annotate models, in order to facilitate their reuse. (2) The Systems Biology Ontology is a set of controlled vocabularies aimed to be used in conjunction with models, in order to characterise their components. (3) BioModels Database is a resource that allows biologists to store, search and retrieve published mathematical models of biological interests. We expect that those resources, together with the use of formal languages such as SBML, will support the fruitful exchange and reuse of quantitative models.

## Introduction

Computational Neurosciences, modelling the function of the nervous systems, have been around for decades. By contrast, Computational Neurobiology, aiming to model the behaviour of the neuron, is a more recent discipline, although developing at a steady pace. The rising popularity of Systems Biology increased the general awareness to modelling and simulation of biological processes, formerly a specific field of Theoretical (or Mathematical) Biology. As a consequence, what was once the territory of a small population of specialists is now visited by various actors of biomedical research. In parallel, the formal models used in Neurobiology and Neurophysiology are growing, both in size and complexity, culminating with the *Blue Brain Project *, that aims to simulate realistic brain functions with a supercomputer. A given modeller is therefore less likely to be an expert of all the corners of a quantitative model, whether the biological knowledge or even the mathematical approaches. Finally, the population of modellers can no longer be identified with the tribe of software developers.

This maturity called for a shift of paradigm in the way software tools are developed and used in the community of Computational Neurobiology. The design of standard formal languages to encode models, such as SBML [1], CellML [2], or NeuroML [3], was a first step. Their development actually served modellers in more than one aspect, fostering the creation of an actual community, and helping to shed light on the bottlenecks that precluded the smooth diffusion and reuse of quantitative models. Now that the way has been paved, one needs to walk forward toward the actual reuse and integration of quantitative models. First of all, one needs more automated support to handle formal models. Modellers should not have to fiddle with the gritty details of file formats for instance, or to have to dissect-out a model to understand what it is about. Secondly, now that the syntax problems are taken care of, the community needs to move to the semantics of the models. Indeed, the fact that a model is encoded in a correct format does not guarantee that it is correct, or even that anybody can understand it. The community has therefore to define agreed-upon standards for model generation and curation. Controlled vocabularies must also be designed for annotating models with connections to biological data resource. Finally, one needs to integrate modelling work with the other sources of knowledge, and disseminate the large number of models produced.

## Tackling the syntax: standard machine-readable formats

A prerequisite to model storage and exchange was the use of standard formal languages. As for each standardisation attempt, the challenge was to balance comprehensiveness and usability. The community of modellers would never agree on a single huge standard, able to describe the wide diversity of quantitative models developed in Neurobiology. Moreover, such a standard would be of limited utility. Indeed, a formal description is only useful if it can be decrypted, and the information it contains successfully reused. Therefore, different tools should only exchange models they can handle. Nevertheless, one could want to use the best features of several descriptions. Fortunately, the use of modern technologies such as XML [4] combined with a careful handling of namespaces permits in some extent to concurrently use several standards.

**Figure 1:**
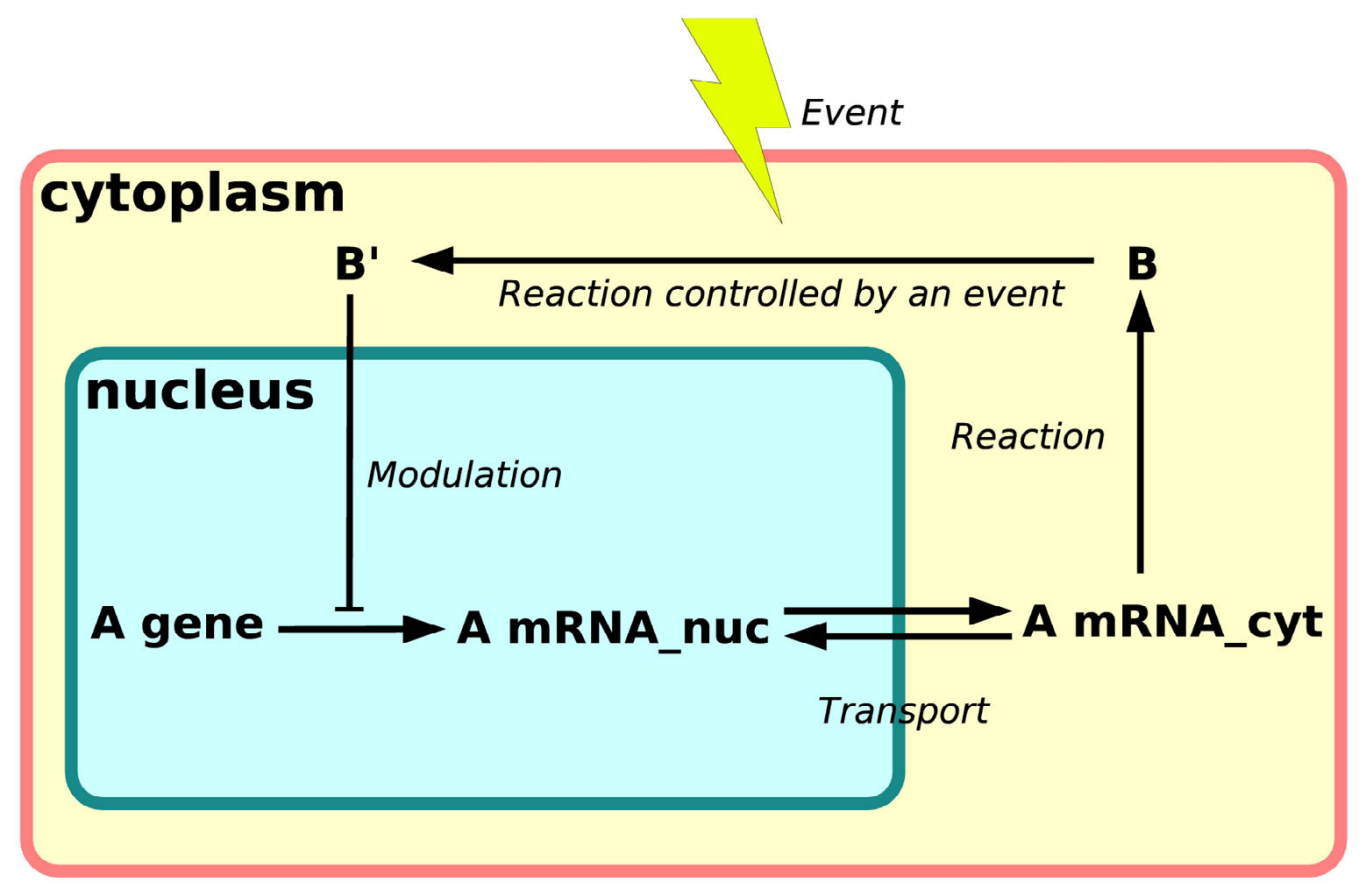
**What can be encoded in SBML?**. Schema representing the type of reactions encoded in SBML, including intra and inter-compartment transformations, modulations, and arbitrary events.

**Figure 2:**
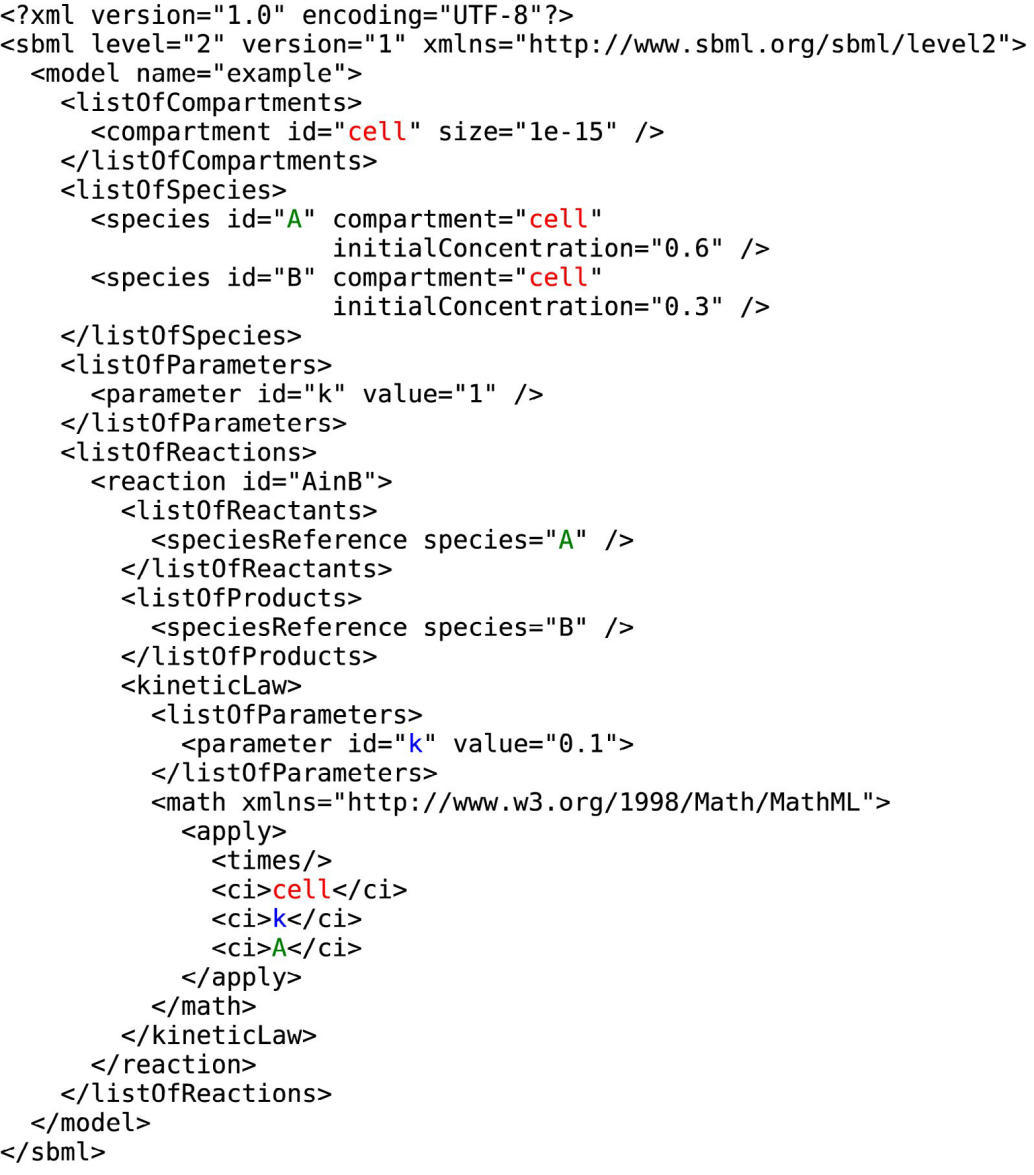
**Minimal example of SBML**. Minimal SBML model, describing the simple conversion of a species A into a species B. The symbols of the same color represent the same numerical value.

Not surprisingly, various formal languages were developed by different communities to encode models at different scales. By far the most successful attempt to develop a language describing quantitative models has been SBML, the Systems Biological Markup Language [1,5]. SBML has been designed for representing models of biochemical reaction networks, but can be used to encode any mechanisms transforming pools of entities according to kinetic laws (, Figure 1, 2). A very similar language is CellML [2]. While the former is based on hierarchical lists of specified elements, the latter describes the model as a collection of linked generic components, thus offering the possibility of modular and multiscale models . SBML is now a community standard, and has even been accepted as a mimetype by the IETF . Its widespread acceptance was fostered firstly because it was designed primarily by its potential users. Secondly, contrarily to other similar languages, its usage is supported by a very precise XML-Schema and a rich library, allowing an easy integration in software based on various programming languages.

NeuroML [3] is a project to develop a series of neuroscience oriented markup-languages . This project is very interesting in the sense that it offers a specification to develop orthogonal although interoperable languages, rather than defining a frozen language. This is very much a prerequisite to encode models ranging from the transduction of neurotransmitter signal to the physiology of micro-circuits. NeuroML is used for instance by the Mesoscopic Reaction Drynamics Simulator .

BrainML  is an effort to provide a standard XML metaformat for exchanging neuroscience data. It focuses on layered definitions to support community-driven extension. Formats to describe biological objects such as *neuron*, *cortex *or *animal model *are available.

Beside the standard formats developed as such by the community, several formalisms initially developed for specific simulators have become *de facto *standards. The best example are the formats of the neuronal simulators GENESIS [6] and Neuron [7].

## Tackling the semantics: what are we talking about?

As Ed Franck, from Argonne National Laboratory, once said about the development of SBML, "The goal is to help people to disagree as precisely as possible". To be able to exchange models under a standard format is completely useless if nobody can interpret the content of the models beside their initial creators. The development and broad acceptance of common model representation formats such as SBML was a crucial step in that direction. The BioModels.net initiative launched in 2004 by Michael Hucka, Andrew Finney and the author is another step: an international effort to (1) define agreed-upon standards for model curation, (2) define agreed-upon vocabularies for annotating models with connections to biological data resources, and (3) provide a free, centralized, publicly-accessible database of annotated, computational models in SBML and other structured formats.

### Minimal information requested in the annotation of models

If searching for existing relevant models, a researcher comes after a model *Model1 *describing the reactions *A *and *B *between the molecular components *X *and *Y*, what can he/she makes of it? Where does this model come from? What are the components *X *and *Y*? It could help to know what process is modelled by *A *and *B*. Providing one finally elucidates the origin of the model, and the identity of its components, how can we know that when instantiated, this model provides the correct numerical results?

The aim of MIRIAM [8] is to define processes and schemes that will increase the confidence in model collections and enable the assembly of model collections of high quality. The first part of the guidelines is a standard for reference correspondence dealing with the syntax and semantics of the model. A second part is a proposed annotation scheme that specifies the documentation of the model by external knowledge. The scheme for annotation can itself be further subdivided into two sections. The *attribution *covers the minimum information that is required to associate the model with a reference description and an actual encoding process. The *external data resources *covers information required to relate the components of quantitative models to established data resources or controlled vocabularies.

The aim of standard for reference correspondence is to ensure that the model is properly associated with a reference description and is consistent with that reference description. The reference description can be a scientific article, but also any other unique publication, on print or online, that describes precisely the structure of the models, list the quantitative parameters, and described the expected output. In order to be declared MIRIAM-compliant, a quantitative model must fulfil the following rules:

1. The model must be encoded in a public, standardised, machine-readable format such as (but not restricted to) SBML or CellML, and it must comply with the standard in which it is encoded.

2. The model must be clearly related to a single reference description. If a model is derived from several initial reference descriptions, there must still be a reference description that describes or references a set of results that one can expect to reproduce when simulating the derived/combined model.

3. The encoded model structure must reflect the biological processes listed in the reference description (a one-to-one correspondence between model components is not required).

4. Quantitative attributes of the model, such as initial conditions and parameters, as well as kinetic expressions for all reactions, have to be defined, in order to allow to instantiate a simulation.

5. The model, when instantiated within a suitable simulation environment, must be able to reproduce all results given in the reference description that can readily be simulated.

In order to be confident in re-using an encoded model, one should be able to trace its origin, and the people who were involved in its inception. The following information should always be joined with an encoded model:

• The preferred name of the model, in order to facilitate discussions about it.

• A citation of the reference description with which the model is associated, either as a complete bibliographic record, or as a unique identifier, Digital Object Identifier , PubMed identifier , unambiguous URL [9] pointing to the description itself etc.

• Name and contact information for the creators who actually contributed to the encoding of the model in its present form.

• The date and time of creation, and the date and time of last modification.

• A precise statement about the terms of distribution. The statement can be anything from "freely distributable" to "confidential". MIRIAM being intended to allow models to be communicated better, terms of distribution are essential for that purpose.

The aim of the external data resources annotation scheme is to link model constituents to corresponding structures in existing and future open access bioinformatics resources. Such data resources can be, for instance, database or controlled vocabularies. This will permit the identification of model constituents and the comparison of model constituents between different models, but also the search for specific constituents in models.

This annotation must permit us to unambiguously relate a piece of knowledge to a model constituent. The referenced information should be described using a triplet {"data-type", "identifier", "qualifier"}. The "data-type" is a unique, controlled, description of the type of data, written as a Unique Resource Identifier [10] (whether a Uniform Resource Locator [9] or a Uniform Resource Name [11]). The "identifier", within the context of the "data-type", points to a specific piece of knowledge. The "qualifier" is a string that serves to refine the relation between the referenced piece of knowledge and the described constituent. Example of qualifiers are "has a", "is version of", "is homologous to", etc. Such a triplet can easely be exported later using RDF [12], to ease further automatic treatment.

To enable interoperability, the community will have to agree on a set of standard valid URIs. and an API should be created so that a tool can automatically retrieve valid URL(s) corresponding to a given URI. The list should be able to evolve with the evolution of data resources.

Whilst many controlled vocabularies exist that can be used to annotate quantitative models, several additional small controlled vocabularies are required to enable the systematic capture of information in those models. This is why BioModels.net partners started to develop their own ontology.

### Systems biology ontology

An ontology is defined here in its information science meaning, as a hierarchical structuring of knowledge. In our case, it is a set of relational vocabularies, that is a set of terms linked together. Each term has a definition and a unique identifier. The most famous ontology in life-science is Gene Ontology (GO) [13]. One of the goals of the Systems Biology Ontology (SBO, ) is to facilitate the immediate identification of the relation between a model component and the model structure. SBO is currently made up of four different vocabularies. Within a vocabulary, the terms are related by "is a" inheritances, which represent sub-classing.

1. A classification of rate laws. This CV is a taxonomy of kinetic rate equations. Examples of terms in this CV are "mass action kinetic", "Henri-Michaelis-Menten kinetics", "Hill function" etc. Note that although taking the same mathematical form, the rate-laws "Henri-Michaelis-Menten", "Van Slyke" and "Briggs-Haldane", being based on different assumptions, will be represented by different terms. This will help a user to choose the adequate conversion to elementary steps if needed.

2. A taxonomy of the roles of reaction participants, including the following terms: "catalyst", "substrate", "competitive inhibitor", "non-competitive inhibitor" etc.

3. A CV for parameter roles in quantitative models. This CV includes terms such as "forward unimolecular rate constant", "Hill coefficient", "Michaelis constant" etc.

4. A list of modelling framework, that precises how to interpret a mathematical expression, such as "deterministic", "stochastic", "boolean" etc.

**Figure 3:**
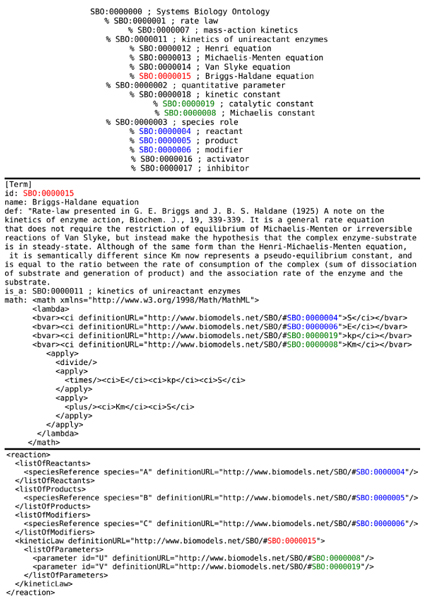
**Integration of SBO and SBML**. *Upper panel*, example of a possible extract of SBO, needed to annotate an enzymatic reaction modeled following Briggs-Haldane steady-state hypothesis. *Middle panel*, the SBO term of the rate-lawbranch describing the Briggs-Haldane kinetic, with its definition and its mathematical expression. *Lower panel*, annotation of an SBML Briggs-Haldane reaction with relevant SBO terms, using the attribute definitionURL introduced in SBML Level2 Version2.

The annotation of model components with SBO terms will be an essential step to reach MIRIAM-compliance. Not only such an annotation will be important to understand and to programmatically analyse models, it will also power the search strategies used by the databases of models, and in particular BioModels Database. The use of SBO terms within SBML will allow to a limited extend to get rid of the explicit mathematics in the model itself, but to download the adequate rate-law instead (Figure 3).

## Data integration and databases

As for all types of knowledge, quantitative models will be only as useful as their access and reuse is easy for all scientists. Several general repositories of quantitative models have been set up. JWS Online [14] is one of the first resources offering curation of the models it distributes, and online simulation. It is linked to the journals *Microbiology*, *FEBS Journal *(former *European Journal of Biochemistry*) and *IEE Systems Biology*, that deposit the models upon submission of the manuscripts, so as to make them available to the reviewers. It now distributes the models in SBML format. The CellML repository [15] distributes models of biochemical and cellular processes encoded in the CellML format. The models cover a wide range of biochemical and cellular processes. The impact of the resource is currently limited by the poor CellML support in the field of kinetic modelling.

The fields of neuronal signalling and electrophysiology have been experiencing model exchange for longer than most other domains. The Database of Quantitative Cellular Signalling (DOQCS) is a repository of models of signalling pathways present in the neurons [16]. It includes reaction schemes, concentrations, rate constants, as well as annotations on the models. The database provides a range of search, navigation, and comparison functions. The pathways can be downloaded in the format used by the neuronal simulator GENESIS [6]. ModelDB  is a database developed as part of the SenseLab effort. The resource distributes models encoded in the many different formats, mainly those used by the GENESIS and NEURON simulators, but also format used by generic simulation environments such as Octave, MatLab, Octave or XPP-Aut. SigPath [17] is an interesting project to develop an open knowledgebase of qualitative pathways and quantitative models related to signalling. An interesting feature is the possibility of annotating model components. As of September 2005, the computing infrastructure is present, but the content of the resource is minimal.

### BioModels database

BioModels Database  is an annotated resource of quantitative models of biomedical interest developed in collaboration by the EMBL-EBI (United-Kingdom), the SBML Team (USA), the Systems Biology Group of the Keck Graduate Institute (USA), the Systems Biology Institute (Japan) and JWS Online at the Stellenbosch University (South Africa). Models can be submitted by anyone to the curation pipeline of the database. At present, BioModels Database aims to store and annotate models that can be encoded with SBML and CellML. BioModels Database goes further than MIRIAM, requiring not only the existence of a reference description, but considering only models described in the peer-reviewed scientific literature. A series of automated tasks are performed by the pipeline prior to human intervention:

• Verification that the file is well-formed XML.

• If necessary, conversion to the latest version of SBML.

• Verification of the syntax of SBML.

• Series of consistency checks, enforcing the validity of the model.

If any of those steps is not completed, a member of the distributed team of curators can reject the model, or instead correct it and resubmit it to the pipeline. The last, and most important step, of the curation process, is verifying that when instantiated in a simulation, the model provides results corresponding to the reference scientific article. Once the model is verified to be valid SBML, and to correspond well to the article, it is accepted in the production database for annotation.

**Figure 4:**
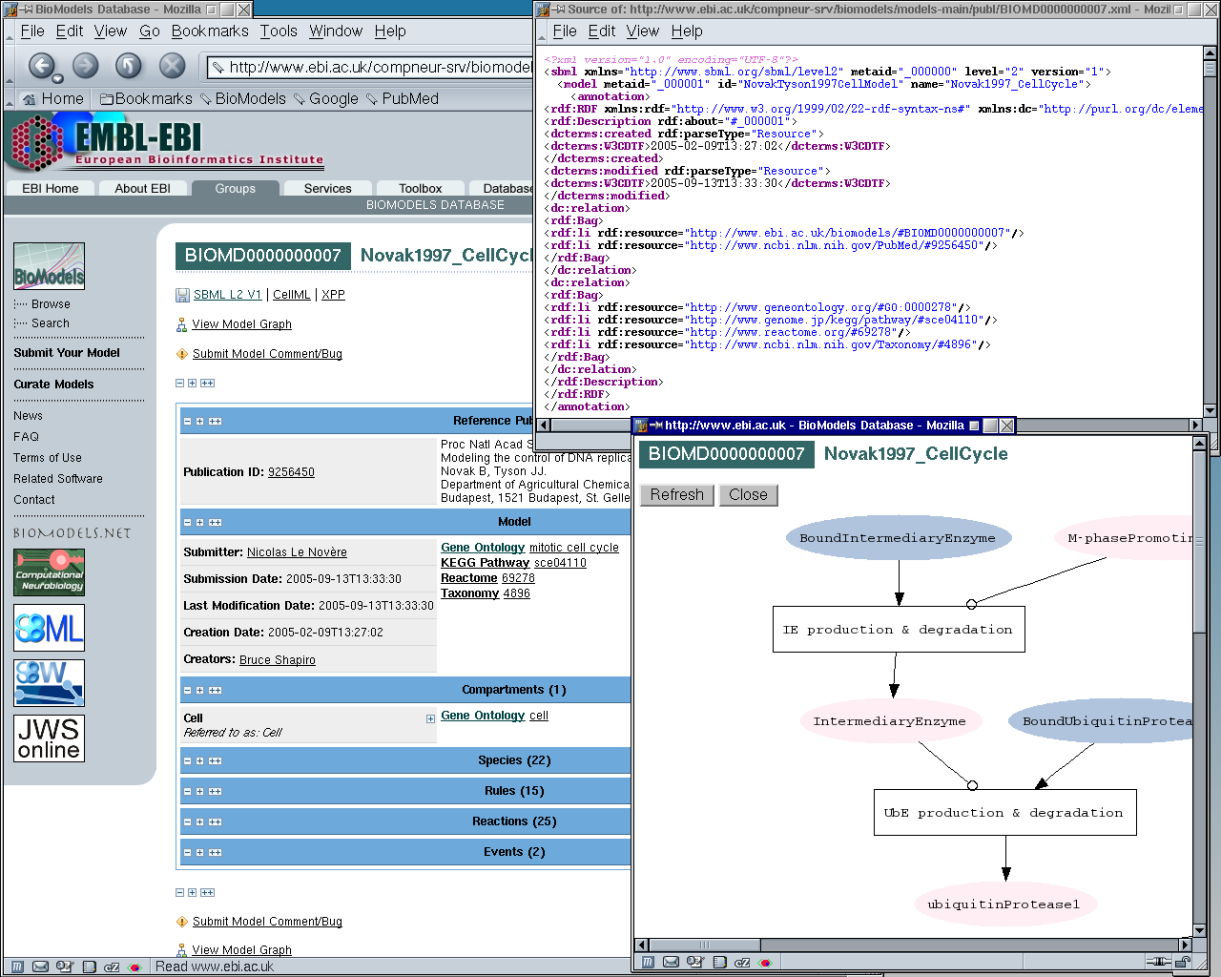
**BioModels Database**. Screenshot showing the entry corresponding to a model of cell cycle [19]. The main page list the components of the model and display the annotations. The reaction graph and the SBML version of the model are shown in different windows.

Model components are annotated with references to adequate resources (Figure 4), such as terms from controlled vocabularies (Taxonomy, Gene ontology, ChEBI etc.) and links to other databases (UniProt, KEGG, Reactome etc.). This annotation is a crucial feature of BioModels Database that permits the unambiguous identification of molecular species or reactions and is used in search strategies. All the annotation is exported in the SBML versions of the models using [12]. The relationships between the model components and the annotation is described using the Dublin Core terms [18]. As a consequence, those models become part of the "semantic web", and the annotation can be easely processed by third party software.

The thorough annotation of models allows a three way search strategy to be run in order to retrieve models of interest. Since the models encoded in SBML are stored directly in an XML native database, those models can be retrieved based on the content of their elements and attributes, using XPath. Models can be retrieved by searching directly the annotation database, using SQL. Although this search is quick, it requires the knowledge of the exact identifiers used by curators to annotate the model. A more advanced search system has therefore been implemented, using direct string search of the third party resources, retrieval of the relevant identifiers, and then search BioModels database for the models annotated with those identifiers. As a consequence, the user can retrieve all the models dealing with "cell cycle" or "MAPK", without having to type "GO:0007049" or "P27361". Once retrieved, the models of interest can be downloaded in SBML Level2, CellML, or as configuration files for various simulator such as XPP-Aut or SciLab. A number of export filters are under development to distribute the models in a wider range of formats.

Although BioModels database is a very recent resource, it has already gained momentum thanks to the support of the SBML community, but also of major scientific actors such as Nature Publishing Group, who publicised its launching and started to submit models. The growth of BioModels Database is currently limited by the curation workforce. It is expected that the existence of a public resource will contribute to improve the quality of the models produced, by putting peer-pressure on the modellers.

## Perspectives

The development of standards to encode and exchange models is a new development in the field of modeling in Neurobiology, mainly driven by the general endorsement of Systems Biology. The consequences of this movement for the field are multiple. First, they make possible the storage and exchange of quantitative models developed using different approaches and tools, while the populations using different software were formerly isolated. A secondary effect of the interoperability is the formation of a community of modellers, who, despite working on different biological processes, can exchange problems and solutions. And finally, the standardisation now permits a stricter evaluation of the validity of the models and their outcome, something that relied largely on trust and blind faith before. We expect that in the long run, the effect will be beneficial and we will witness a gradual increase in the quality and the usefulness of the quantitative models of neuronal function.
